# Comparison of Trueness of Digitally‐ and Conventionally‐Fabricated Mockups

**DOI:** 10.1002/cre2.70307

**Published:** 2026-02-10

**Authors:** Mehran Falahchai, Mahyar Ezzati, Amirreza Hendi

**Affiliations:** ^1^ Department of Prosthodontics, Dental Sciences Research Center, School of Dentistry Guilan University of Medical Sciences Rasht Iran; ^2^ Department of Prosthodontics, School of Dentistry Islamic Azad University of Tehran Medical Sciences Tehran Iran

**Keywords:** computer‐aided design, printing, stereolithography, three‐dimensional

## Abstract

**Objectives:**

Rising demands for aesthetic outcomes make precise preoperative assessment essential. Despite advances in digital workflows, their accuracy versus conventional techniques remains uncertain. The current study comparatively assessed the trueness of digitally‐ and conventionally‐fabricated mockups.

**Materials and Methods:**

Fourteen patients needing ceramic veneers in the anterior maxilla participated. Each received three mockups: one fabricated conventionally and two digitally (additive and subtractive). The mockups were placed intraorally and scanned. The scans were superimposed on a reference model to evaluate the accuracy of the entire workflow and on the reference wax‐up to assess production accuracy. Data were analyzed by paired samples test, repeated measures ANOVA, Bonferroni test, and generalized estimating equation (GEE) (*α* = 0.05).

**Results:**

For the whole production process, the root mean square (RMS) was 0.13 (0.04) for the conventional method, which was significantly lower than that for the additive as 0.60 (0.07) and subtractive of 0.51 (0.07) digital techniques (*p* < 0.001). Also, for the production phase, the mean RMS of different methods was significantly different (*p* < 0.001). The conventional method showed the lowest mean by 0.22 (0.07), and the additive technique showed the highest mean by 0.76 (0.02) RMS. The mean RMS was 0.73 (0.02) for the subtractive method.

**Conclusions:**

The trueness of the conventional method was higher than that of the digital method for both the whole production process and the production phase. The conventional method significantly decreases the chairside time especially when tooth surface treatment is not required. The trueness of the subtractive method was higher than that of the additive method.

## 1 Introduction

In modern dentistry, patient demands for esthetic outcomes have significantly increased, requiring that cosmetic procedures closely align with patient expectations (Malmqvist et al. 2024). Diagnostic wax‐ups and mockups are routinely used to predict treatment results and guide tooth preparation (Moldovani et al. 2023; Villalobos‐Tinoco et al. 2022). Mockups also facilitate communication among the clinician, technician, and patient, support conservative enamel reduction, and contribute to predictable biomechanics and esthetics (Osorio‐Vélez et al. 2024). Digital techniques can reduce fabrication time, material waste, and procedural errors (Ijaz 2024). In contrast, conventional methods are more technique‐sensitive and may involve issues such as misplacement of the silicone matrix, uneven pressure during polymerization, and difficulties in removing excess material (Cattoni et al. 2019).

Digital wax‐ups can be produced by subtractive or additive manufacturing. Computer‐aided design and manufacturing (CAD‐CAM) systems—including a scanner, CAD software, and CAM milling unit—enable high accuracy and reproducibility for subtractive techniques; however, milling vibrations may reduce trueness (Kim et al. 2016, 2017). Additive techniques, such as three‐dimensional (3D) printing, have advanced restorative workflows through rapid prototyping and automation (Tahayeri et al. 2018). Restorations can be fabricated from various materials including composite resins, ceramics, zirconia, and polymethyl methacrylate (PMMA), which is valued for its biocompatibility, hardness, cost‐effectiveness, and ease of use (Arcuri et al. 2018; Zafar 2020).

Although previous studies have assessed the accuracy of various fabrication methods for provisional and definitive restorations (Cho et al. 2015; Kang et al. 2018; Tahayeri et al. 2018), few have comprehensively evaluated the entire mockup process. Because inaccuracies in mock‐up fabrication may originate either from the manufacturing technique itself or from cumulative clinical and procedural steps, evaluating trueness at two levels is essential. The production‐phase analysis isolates the accuracy of the fabrication method (conventional, additive, or subtractive), while the entire‐workflow analysis reflects the overall deviation after intraoral placement and clinical handling. Comparing both provides a more complete understanding of the sources of error within the mock‐up workflow. This study aimed to assess the trueness of mockups fabricated by conventional, additive, and subtractive techniques, considering all clinical steps from production to intraoral placement. The null hypothesis was that all three methods would show similar trueness for both the complete workflow and the production phase.

## 2 Materials and Methods

### 2.1 Study Design

This study included 14 patients requiring ceramic veneers in the maxillary anterior region (canine to canine) who presented to the Prosthodontics Department. The minimum required sample size was calculated as 13 using PASS 11 software, based on an expected effect size of 0.90, study power of 0.99, a significance level of 0.05, and allowing for a 20% dropout rate (Jeong et al. 2018). Inclusion criteria were adult patients (> 18 years) in good systemic and mental health (ASA I or II) requiring smile design correction with ceramic veneers in the canine‐to‐canine area and not needing additional tooth surface treatment (Gonzalez‐Martin et al. 2021). Exclusion criteria included periodontal disease, pregnancy, or allergy to resin materials (Duggal et al. 2021; Memon et al. 2022; Gonzalez‐Martin et al. 2021; Zur 2023). Written informed consent was obtained for use of patient photographs and for participation in the restorative procedures.

### 2.2 Mockup Fabrication Process

**Figure 1:**
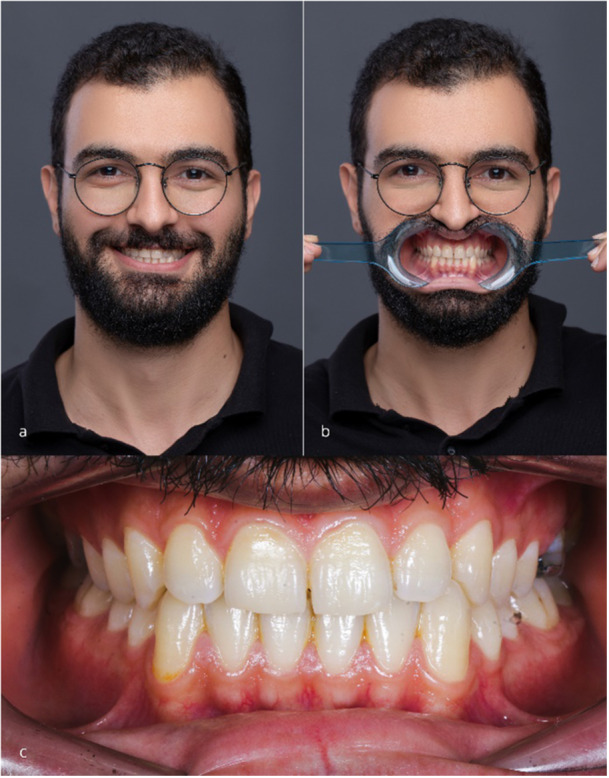
Initial photographs required for the conventional and digital smile design: (a) Frontal smile view, (b) frontal small view with retracted lips, (c) intraoral photograph of the patient in maximum intercuspation.

For each patient, mockups were fabricated using both a fully digital workflow (additive and subtractive) and a conventional method. The resulting scans were used to assess trueness. Diagnostic records included extraoral photographs (maximum smile and retracted lips) and intraoral photographs taken at the first appointment (Figure 1) (Gadallah et al. 2023). Primary impressions were made conventionally using a one‐step putty and light‐body polyvinyl siloxane (Panasil, Kettenbach GmbH and Co. KG, Germany) and digitally using a full‐arch intraoral scanner (Planscan; Planmeca USA Inc.). Conventional impressions were poured with type IV die stone (GC Fuji Rock EP; GC Europe, Leuven, Belgium).

**Figure 2:**
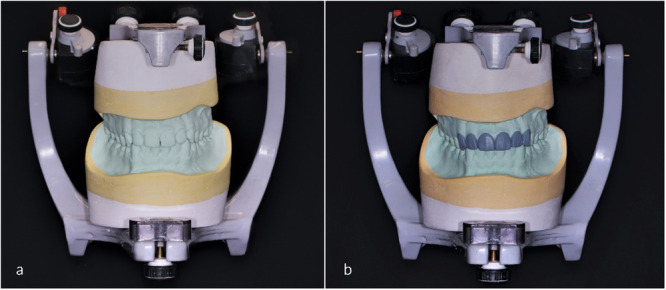
(a) Casts mounted on a semi‐adjustable articulator; (b) laboratory wax‐up.

The stone casts were mounted on a semi‐adjustable articulator (Archimedes PRO Articulator Arcon Semi‐Adjustable, MESTRA Manufacturing Co., Bilbao, Spain) using elastomeric bite records (Futar D, Kettenbach GmbH and Co. KG, Germany) (Figure 2). A conventional diagnostic wax‐up was completed for each case based on clinical instructions and photographs. Wax‐ups were scanned with an intraoral scanner to serve as a reference for evaluating the production phase trueness. Patients returned for mockup placement and approval of the final form.

A putty index (Panasil) of the wax‐up was used to fabricate mockups from a self‐cure resin material (Structur 2, Voco GmbH, Germany). Before adjustments, conventional mockups were scanned to evaluate trueness for both the production phase and the complete process. Final modifications were made according to anatomical landmarks, lip support, and patient feedback. A final full‐arch maxillary scan was then obtained with an intraoral scanner to serve as the reference for assessing the trueness of the entire production process for both conventional and digital methods.

### 2.3 Digital Fabrication Process

After removal of the conventional mockup material, the digital mockup fabrication process was initiated. The STL files containing the primary scans of both jaws and the bite registration were imported into CAD software (Exocad DentalCAD; Exocad GmbH). A digital wax‐up was designed directly on the scan model. This digital wax‐up served as the reference for evaluating the trueness of the additive and subtractive production phases (but not the entire production process).

**Figure 3:**
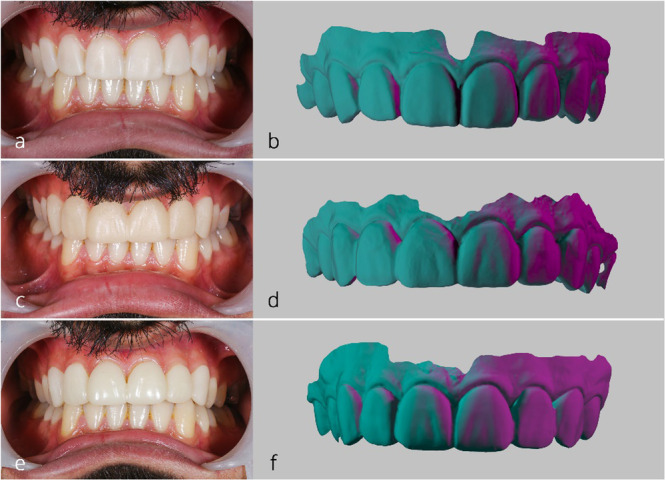
Fabricated mockups prior to any intraoral adjustment: (a) Conventional mockup; (b) conventional mockup scan, (c) subtractive mockup, (d) subtractive mockup scan, (e) additive mockup, and (f) additive mockup scan.

The digital wax‐up was used to fabricate mockups with two techniques: one using a methacrylate oligomer resin (C&B MFH Composite; NextDent by 3D Systems) printed with a 3D printer (Asiga; Australia) employing digital light processing (additive method), and one using polymethyl methacrylate resin blocks (ZCAD Temp Fix 98; Harvest Dental) milled with a milling machine (Ceramill, Amann Girrbach, Austria) (subtractive method). Additive mockups were printed with a 45° orientation and a 50 µm layer thickness (Espinar et al. 2023; Gao et al. 2021; Topsakal et al. 2023). Printed mockups were cleaned in 90% ethanol for 5 min, air‐dried, and post‐polymerized in a UV curing unit (LC‐3DPrint Box; NextDent by 3D Systems) for 30 min. Finally, the digital mockups were seated on the teeth and scanned with an intraoral scanner for further trueness assessment (Figure 3). All intraoral scans, primary scans, digital mock‐up scans after intraoral placement, and the final full‐arch reference scan, were obtained using the same intraoral scanner (Planscan; Planmeca USA Inc).

### 2.4 Trueness of Mockups

**Figure 4:**
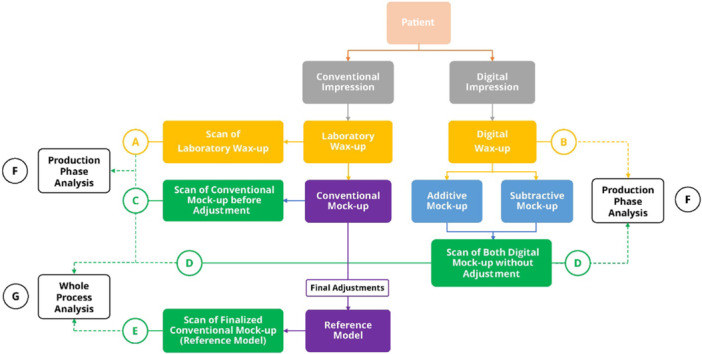
(A) Conventional impressions underwent laboratory wax‐up and scanning. (B) Digital impressions were transferred to CAD software for wax‐up. (C) Laboratory wax‐up was used to fabricate a conventional mockup, which was scanned prior to any intraoral adjustment. (D) Additive and subtractive mockups fabricated from a digital wax‐up were placed in the oral cavity, and scanned without any intraoral adjustment. (E) After final adjustment of the conventional mockups (reference models), they were scanned. (F) To analyze the production phase, the conventional mockup scans were compared with their laboratory wax‐up scan and the digital mockup scans were compared with their design file. (G) To analyze the whole production process, the conventional and digital mockup scans were compared with the reference model scan.

To evaluate the impact of the entire production process on mockup trueness, all mockup scan files per patient, including digital mockups and the conventional mockup before final adjustment, together with the reference scan were imported into reverse‐engineering software (Geomagic Control X 2018.1.1.51, 3D Systems, Cary, USA). For the whole‐process trueness analysis, the reference model corresponded to the fully adjusted esthetic mockup, which had undergone intraoral refinement according to smile‐esthetic and functional criteria (lip dynamics, gingival display, tooth proportions, occlusal harmony, and patient feedback). Importantly, the scan used for comparison in the conventional group was obtained before these final adjustments; therefore, the subsequent esthetic refinement, scanning procedures, and alignment steps contributed to the deviation of the conventional workflow as well. The same clinically validated reference model was used for all groups to allow standardized whole‐process comparison. To isolate the production phase effect, scans of mockups prior to any adjustment were compared with their respective reference scans. For the conventional technique, the reference was the scanned conventional wax‐up, whereas for digital methods, the digital wax‐up design file was used. Figure 4 schematically illustrates the study design.

### 2.5 Data Assessment

**Figure 5:**
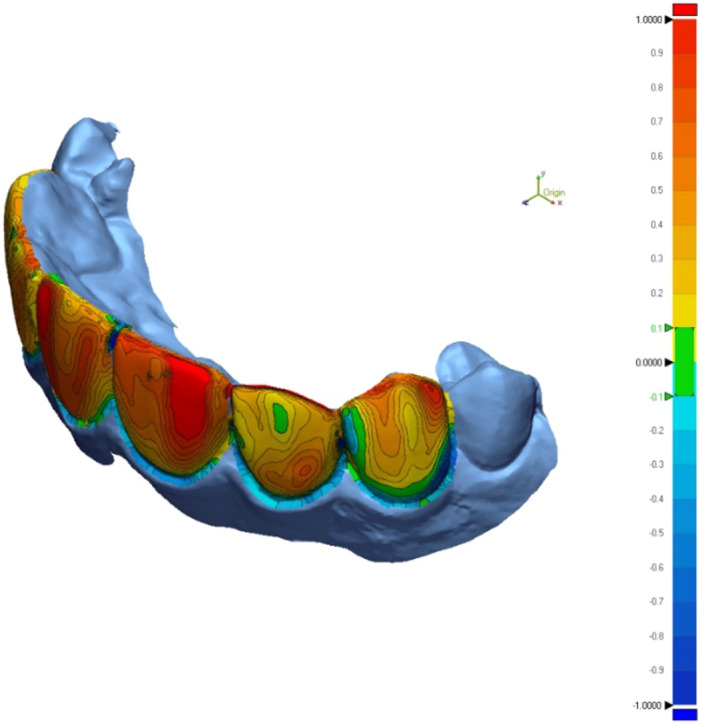
Color map of the comparison of the conventional mockup with the reference model (to assess the trueness of the whole production process).

Qualitative analysis involved superimposition of mockup scans with their references using the Best Fit Alignment tool, followed by color mapping of deviations (Figure 5). Color map limits were set at +100 µm and −100 µm, with an acceptable deviation range defined between −30 µm and +30 µm. Quantitatively, root mean square (RMS) deviation was calculated from the superimposed scans using the formula (Metin et al. 2024):

$$\mathrm{RMS}=\sqrt{\frac{\sum_{i=1}^{n}{\left(x_{1,i}-x_{2,i}\right)}^{2}}{n}}$$

where *x* ^1^,*i* ^*x*^_{1,*i*}*x* ^1^,*i* represents the reference data points, *x* ^2^, *ix*_{2,*i*}*x* ^2^,*i* the corresponding scan data points, and *n* the total number of measured points. This metric objectively quantifies the accuracy and geometric fit of the specimens.

### 2.6 Statistical Analysis

Data normality was assessed via the Shapiro–Wilk test, and variance homogeneity via Levene's test. Data were reported as mean (SD) and statistical comparisons employed paired samples tests, repeated measures ANOVA with Bonferroni‐corrected pairwise comparisons, and generalized estimating equations (GEE) with an unstructured working correlation matrix. The Quasi Likelihood under Independence Model Criterion (QIC) informed model selection. All analyses were performed using SPSS version 26 with a significance level of 0.05.

## 3 Results

For the production phase analysis, the reference was the conventional wax‐up scan for the conventional mock‐ups and the digital design file for the additive and subtractive mock‐ups. For the whole process analysis, the final full‐arch maxillary scan (obtained after adjustment of the conventional mock‐up) was used as the common reference model for all three fabrication methods. Table 1 summarizes the trueness results of mockups fabricated using digital and conventional techniques across the entire production process. Repeated measures ANOVA comparing RMS values and GEE assessing changes among methods revealed significant differences in mean RMS across fabrication techniques (*p* < 0.001). The conventional method demonstrated the lowest RMS (0.22 [0.07]), while the additive method had the highest (0.76 [0.02]). Similarly, RMS values differed significantly among groups for the fabrication phase, with the conventional group showing the lowest mean RMS (0.13 [0.04]) and the additive group the highest (0.60 [0.07]) (Table 1). Pairwise comparisons using the Bonferroni test indicated significant differences across all groups for both the production phase and the entire process (Table 2). In this analysis, the final full‐arch maxillary scan served as the reference model for superimposition in all groups (conventional, additive, and subtractive), and the calculated RMS values are reported in the “whole process” column of Tables 1 and 2.

**Table 1:** Comparison of the trueness/accuracy of mockups fabricated by the conventional and digital (subtractive and additive) techniques for the production phase and the whole production process.

	Production phase			Whole process		
	Mean (SD) (mm)	*p‐*value*	Statistic	Mean (SD) (mm)	*p*‐value*	Statistic
Subtractive	0.73 (0.02)	< 0.001	244.04	0.51 (0.07)	< 0.001	904.26
Additive	0.76 (0.02)			0.6 (0.07)		
Conventional	0.22 (0.07)			0.13 (0.04)		

* Repeated measures.

**Table 2:** Pairwise comparison of the methods.

	Production phase*			Whole process*		
	Conventional	Additive	Subtractive	Conventional	Additive	Subtractive
**Conventional**	—	—	—	—	—	—
**Additive**	< 0.001	—	—	< 0.001	—	—
**Subtractive**	< 0.001	< 0.001	—	< 0.001	0.008	—

* Bongerroni test.

Qualitative analysis indicated that both conventional and digital mockups tended to be bulkier than the reference model, particularly in buccal and embrasure regions. Notably, digitally fabricated mockups, especially those produced via the additive technique, exhibited greater thickness at marginal areas compared to conventional mockups.

## 4 Discussion

This study employed a comprehensive approach to compare the trueness and reproducibility of conventional, additive, and subtractive mockup fabrication methods, providing insight into digital versus conventional workflows. Clinical relevance was emphasized by assessing mockup accuracy directly on patients' teeth, enhancing the applicability of results to real‐world settings. The combined use of qualitative (color mapping) and quantitative (RMS) analyses offered a multidimensional evaluation of accuracy and fit, supporting the robustness of the findings. Additionally, the study utilized a non‐destructive 3D conformance method for adaptation assessment across the three fabrication techniques. Results indicated variability in trueness among methods, leading to rejection of the null hypothesis. Direct dental mockups prior to preparation allow clinicians to visualize smile design modifications in situ, facilitating adjustments in veneer length, shape, or spacing according to lip line and esthetic parameters before definitive restoration fabrication (Jafri et al. 2020). To ensure sample standardization and avoid irreversible procedures, only patients requiring additive treatments were included, while those needing subtractive preparation prior to mockup fabrication were excluded; thus, no surface treatment was necessary as an inclusion criterion.

An important component of the present investigation is the whole‐process trueness assessment, which showed that the conventional workflow achieved the lowest cumulative deviation, followed by the subtractive and additive digital techniques. No previous study has assessed the cumulative deviation of the entire mockup fabrication workflow against a clinically validated esthetic endpoint. In our design, the reference model for whole‐process analysis corresponded to the fully adjusted esthetic mockup, refined intraorally according to smile‐esthetic and functional criteria. Thus, the reference did not represent the unmodified output of the conventional workflow but rather the finalized clinical form that constitutes the intended treatment outcome and could theoretically be reached using any fabrication method. The non‐zero whole‐process deviation in the conventional group confirms that its comparison scan was obtained before final intraoral adjustments, meaning that clinical refinement, scanning variability, and alignment procedures contributed to its measured discrepancy.

Whole‐process trueness therefore answers a clinically meaningful question, namely, how accurately each fabrication workflow reproduces the final esthetic and functional outcome accepted in patient care, while the production‐phase analysis isolates the intrinsic manufacturing accuracy of each technique. Together, these complementary analyses provide a comprehensive evaluation of workflow performance that has not been previously presented in the literature. It should also be noted that intraoral scanners inherently introduce a small degree of trueness deviation. However, because the same scanner, operator, and acquisition protocol were used for all mockups, any IOS‐related error would have influenced all workflows similarly and therefore cannot account for the inter‐group differences observed in whole‐process RMS values.

Our results indicated that the conventional method demonstrated greater accuracy than the digital techniques, which may be attributed to inherent limitations of digital workflows in capturing fine details comparable to conventional methods. Wear of burs in subtractive processes can reduce the precision of milled restorations over time (Al Hamad et al. 2021). Additionally, factors such as layer thickness, laser intensity, and print orientation influence the quality of mockups produced by additive manufacturing (de Castro et al. 2022; Tahayeri et al. 2018). Each fabrication technique presents specific material and mechanical constraints: bur wear and variable material hardness can affect milling accuracy, while printer calibration, geometry, and resin properties impact 3D printing outcomes.

Clinical factors such as cementation and oral environment interactions were not simulated in this study. Cementation may better reveal discrepancies in mockup fit, as direct resin mockups can adapt more closely to tooth surfaces before adjustment, whereas digital mockups reflect discrepancies due to the pre‐set cement space (Bi et al. 2022; Binali et al. 2023; Klauer et al. 2020; Moayyedian et al. 2020). Prior research has shown that digital mockups, particularly those produced subtractively, may offer improved accuracy and efficiency compared to conventional methods when used for crowns and temporary restorations where fit and esthetics are critical. Evidence reported that digital mockups scored higher in proportional accuracy and arch form, and provided esthetic benefits, despite limitations in surface quality likely related to milling constraints. Although the additive technique did not surpass subtractive accuracy, it offers greater flexibility for complex geometries and cost‐effectiveness, making it suitable for prototyping or applications where design complexity outweighs the need for maximal accuracy (Chisnoiu et al. 2023; Huang et al. 2022; Javaid and Haleem 2019).

Few studies have directly compared different mockup fabrication methods; however, relevant findings from studies comparing digital and conventional techniques for other dental restorations warrant discussion to contextualize conflicting results (Koh et al. 2025; Manisha et al. 2023; Souto et al. 2024). Souto et al. (2024) reported that the conventional technique provided greater accuracy than digital impressions for fixed partial dentures, highlighting the adaptability of conventional methods. While subtractive techniques show promise, they may not fully replace conventional methods for restorations requiring high precision. These findings align with the present study's results, which demonstrated superior accuracy and finer detail in conventionally fabricated mockups compared to those produced via additive or subtractive digital methods (Ellakany et al. 2022; Koh et al. 2025). Abduo et al. (2023) reviewed the accuracy of digital fabrication for zirconia crowns and noted variability depending on environmental factors and treatment protocols. Intraoral challenges such as saliva presence and limited space can reduce scan accuracy (Sindhu et al. 2023). These variables may explain discrepancies between digital and conventional methods, as conventional techniques tend to be less affected by such clinical conditions.

Regarding digital techniques, Lee et al. found that subtractive zirconia crowns generally exhibit better internal adaptation than additive crowns, although additive crowns had superior marginal adaptation. Overall, subtractive manufacturing demonstrated higher accuracy than additive methods (Lee et al. 2024). Similarly, Çakmak et al. (2024) concluded that both additive and subtractive techniques are clinically acceptable for crown fabrication, though additive restorations may require longer chairside adjustment. Their results are consistent with the current findings, showing greater accuracy of subtractive over additive mockups, albeit both being less accurate than conventional mockups (Koh et al. 2025).

Variations in reported outcomes likely stem from differences in restoration types, device configurations, materials, and operator skills, all affecting digital fabrication performance. These inconsistencies highlight the need for controlled studies to identify which digital methods can reliably replace conventional techniques. The limited number of studies focused on mockups indicates a research gap, possibly due to rapid technological advances. Future research should evaluate esthetic results, accuracy, and cost‐effectiveness to guide clinical decisions. Additionally, further studies including tooth preparation, cementation, and intraoral adjustments are warranted. Comparisons across multiple 3D printer and milling machine brands, with more operators, are needed to minimize errors.

In the whole‐process analysis, a single clinically adjusted mockup was used as the common reference model. While this reflects the esthetic and functional endpoint typically accepted in patient care, the use of a universal reference warrants cautious interpretation and should be considered in parallel with the production‐phase analysis, which evaluates the intrinsic accuracy of each fabrication technique using its own design reference. Limitations include testing only one brand/model of milling machine, 3D printer, and resin, limiting generalizability. Different devices vary in accuracy, so future studies should include a broader range. The study focused on ceramic laminate veneers; minor software alignment errors may affect accuracy. Thickness limitations of polymethyl methacrylate and digital fabrication restrict producing very thin restorations like mockups. Digital methods may require external contour adjustments, especially at margins, whereas conventional mockups need minimal modification. Finally, trueness is only one factor influencing clinical outcomes. Further in vitro and in vivo research should assess mechanical and optical properties such as wear resistance, fracture toughness, color stability, and translucency to enhance clinical application.

## 5 Conclusion

Based on the present results, the conventional method demonstrated greater overall trueness throughout the entire production process compared to digital methods, particularly in cases requiring little to no tooth surface preparation. Additionally, the conventional approach significantly reduces chairside time for surface and marginal adjustments. Regarding the production phase alone, the conventional method also exhibited higher trueness. Among the digital techniques, the subtractive method achieved greater accuracy than the additive method.

## Author Contributions

Mehran Falahchai contributed to the conceptualization, data curation, formal analysis, investigation, methodology, project administration, software, visualization, and writing of the original draft. Mahyar Ezzati contributed to data curation and investigation. Amirreza Hendi contributed to the conceptualization, methodology, supervision, project administration, and writing by reviewing and editing the manuscript.

## Ethics Statement

The study protocol was approved by the ethics committee of Guilan University of Medical Sciences (IR.GUMS.REC.1399.601).

## Conflicts of Interest

The authors declare no conflicts of interest.

## Data Availability

The datasets used and analyzed in the current study are available from the corresponding author upon reasonable request.
